# DMSO might impact ligand binding, capsid stability, and RNA interaction in viral preparations

**DOI:** 10.1038/s41598-024-81789-x

**Published:** 2024-12-06

**Authors:** Jiri Wald, Nikolaus Goessweiner-Mohr, Antonio Real-Hohn, Dieter Blaas, Thomas C. Marlovits

**Affiliations:** 1 Institute of Microbial and Molecular Sciences, University Medical Center Hamburg-Eppendorf, Hamburg, Germany; 2 Centre for Structural Systems Biology, Hamburg, Germany; 3 Deutsches Elektronen Synchrotron (DESY), Hamburg, Germany; 4 Institute of Biophysics, Johannes Kepler University (JKU), Linz, Austria; 5 Center for Medical Biochemistry, Max Perutz Laboratories, Vienna Biocenter, Medical University of Vienna, Vienna, Austria

**Keywords:** Rhinovirus, HRV, DMSO, Dimethyl sulfoxide, Pocket factor, Cryoelectron microscopy, Virus structures

## Abstract

Dimethyl sulfoxide (DMSO) is a widely used solvent in drug research. However, recent studies indicate that even at low concentration DMSO might cause structural changes of proteins and RNA. The pyrazolopyrimidine antiviral OBR-5-340 dissolved in DMSO inhibits rhinovirus-B5 infection yet is inactive against RV-A89. This is consistent with our structural observation that OBR-5-340 is only visible at the pocket factor site in rhinovirus-B5 and not in RV-A89, where the hydrophobic pocket is collapsed. Here, we analyze the impact of DMSO in RV-A89 by high-resolution cryo-electron microscopy. Our 1.76 Å cryo-EM reconstruction of RV-A89 in plain buffer, without DMSO, reveals that the pocket-factor binding site is occupied by myristate and that the previously observed local heterogeneity at protein–RNA interfaces is absent. These findings suggest that DMSO elutes the pocket factor, leading to a collapse of the hydrophobic pocket of RV-A89. Consequently, the conformational heterogeneity observed at the RNA-protein interface in the presence of DMSO likely results from increased capsid flexibility due to the absence of the pocket factor and DMSO-induced affinity modifications. This local asymmetry may promote a directional release of the RNA genome during infection.

**Supplementary Information:**

The online version contains supplementary material available at 10.1038/s41598-024-81789-x.

## Introduction

Rhinoviruses (RVs) are the main cause of the common cold^1,2^. More than 170 serotypes circulate in the population and cross-immunity is generally weak making vaccination impractical. Nevertheless, efforts toward creating a universal vaccine are continuing^3^. On the other hand, many antivirals have been developed over the last decades but none was approved by the FDA because of side effects and low efficacy^4^; yet, the quest is still ongoing^5^. In general, RV infection is mild and resolves within about a week; medication must thus sensibly shorten the duration of the unpleasant symptoms and be virtually side-effect free to be accepted by the patients.

Belonging to the large family *Picornaviridae*, the typical architecture of RVs comprises a ~ 30 nm diameter capsid enclosing a ss(+) RNA genome of about 7200 bases. The building blocks are four capsid proteins (VPs) present as 60 copies each and arranged on a pseudo T = 3 lattice. After the first 3Dstructures of RV-B14^6^ as well as of the closely related poliovirus type 1^7^ had been solved, high-resolution X-ray and cryo-electron microscopy (cryo-EM) structures of numerous RV types and of closely related members of the *Enterovirus* genus became available and have been used for the design of capsid-binding compounds. These drug candidates attach inside a hydrophobic pocket mainly contributed by amino acid residues of VP1 and displace a natural pocket factor—in most cases a myristate—stabilizing the viral shell against structural changes required for release of the viral RNA genome into the host cell’s cytosol for infection^8^. Some of the 3D-maps of the above RVs lacked a pocket factor altogether or exhibited two alternative conformations of the virion, one with a full, one with an empty pocket^9,10^. This led to the discussion of which one is the natural form of the virion and whether the pocket factor might get lost during viral purification.

During earlier work on the structure of a complex between the pyrazolopyrimidine antiviral OBR-5-340 and RV-B5, RV-A89 that is not inhibited by OBR-5-340, was used as a negative control. Its cryo-EM structure solved in the presence of OBR-5-340 in 10% DMSO showed an empty pocket similar to that in RV-B14^6^ and that in the empty conformation of RV-A16^9^. The authors assumed that the pocket factor was lost during viral purification. Nevertheless, an effect of the DMSO-containing solvent could not be excluded.

Following up on these results we determined the cryo-EM structure of the same RV-A89 in plain buffer lacking OBR-5-340 and DMSO and found that the hydrophobic pocket was filled with myristate. Here, we report on the differences between the two structures. Furthermore, we present the result of a comparison between computationally extracted single protomers; whereas a previous analysis of the 3D map obtained from RV-A89 in presence of DMSO had demonstrated heterogeneity at the protein/RNA interface^11^, in plain buffer, RV-A89 was highly symmetric with single protomers being essentially identical and thus not segregating into different 3D classes. We hypothesize that absence of the pocket factor makes the capsid more flexible, allowing the sixty protomers in the viral capsid to individually interact with the different loops and double stranded regions of the asymmetric RNA more specifically and more closely. However, the observed phenomenon might also be uniquely attributed to the RNA conformation-modifying effect of DMSO^12^.

## Results

### Effect of DMSO on the hydrophobic pocket

Dimethyl sulfoxide (DMSO) is widely used to dissolve small organic compounds in drug-screening experiments including 3D-structure determination; this also applies to antiviral screening studies for rhinoviruses^13^. In previous work Wald and colleagues used DMSO as a solvent for the antiviral compound OBR-5-340 and determined the structure of RV-A89 in the presence of 10% DMSO by using cryo-EM. It was expected that OBR-5-340 was absent from the pocket as RV-A89 is not inhibited by this compound. However, unexpectedly, density, corresponding to a fatty acid, such as myristate, was also absent. As clearly visible and previously discussed^14^, the pocket was collapsed. Thus, we hypothesized that the DMSO in the buffer might be responsible for removing the myristate from the hydrophobic pocket of RV-A89.

**Fig. 1:**
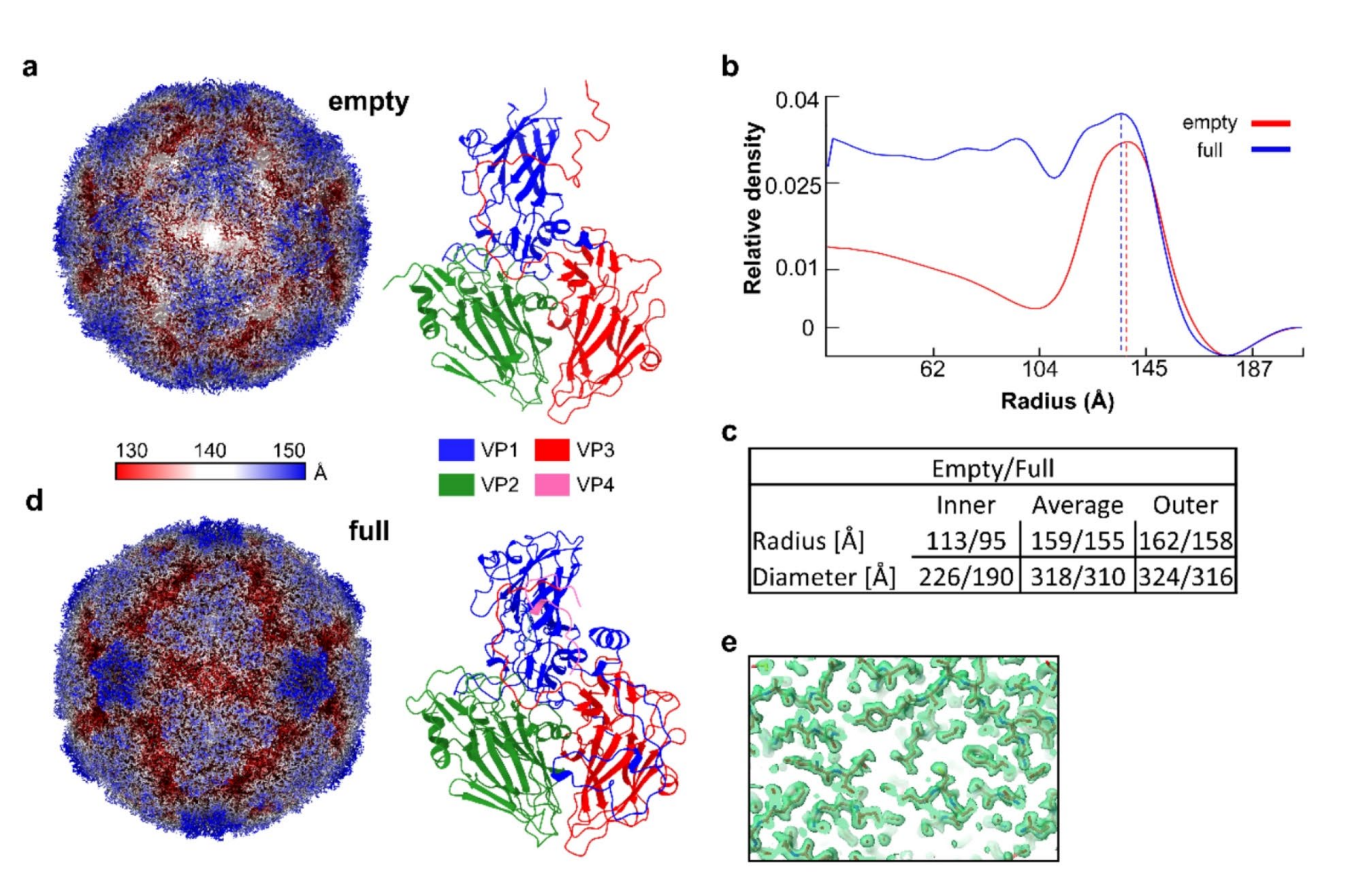
Structures of RV-A89 capsids. Empty and full capsids in the sample of RV-A89 incubated without DMSO. The view is down a twofold axis. (**a**) Structure of the empty RV-A89 capsid missing the VP4 protein. (**b**) Radial density profiles of 3D reconstruction of empty (red) and full (blue) particle. Dashed lines indicate the respective maximum density. (**c**) Dimensions analysis using ViperDB on-line tool^54^. (**d**) Structure of a full RV-A89 capsid. (**e**) Representative example of cryo-EM density and fitted model.

To test this hypothesis and to further investigate the effect of DMSO at the structural level we solved the structure of native RV-A89 in plain (DMSO-free) buffer by high resolution cryo electron microscopy and compared it with the previously published map^14^. During 2D classification of the extracted particle images we also noted that about 20% of the particles represented empty capsids. Following the infection and virus purification protocol^15^, we usually found empty particles in the range of a low two-digit percentage of total viral particles^16,17^. Solving the structure of the RV-A89 empty particles to 2 Å allowed us to build an atomic model and to notice that the VP4 protein was missing (Fig. 1a, Sup. Figure 1a–f). This, together with the capsid diameter analysis that revealed about 2% expansion with respect to native virions (Fig. 1b, c), identify them as B particles^18–20^ that remain after uncoating^21^. Therefore, they are not ‘natural empty capsids (NEC)’ also termed ‘natural top component’ that contain VP0 instead of its maturation products VP2 and VP4^22,23^. These latter particles are believed to either constitute precursors of native virions or dead-end assembly products resulting from defective encapsulation of the RNA.

**Fig. 2:**
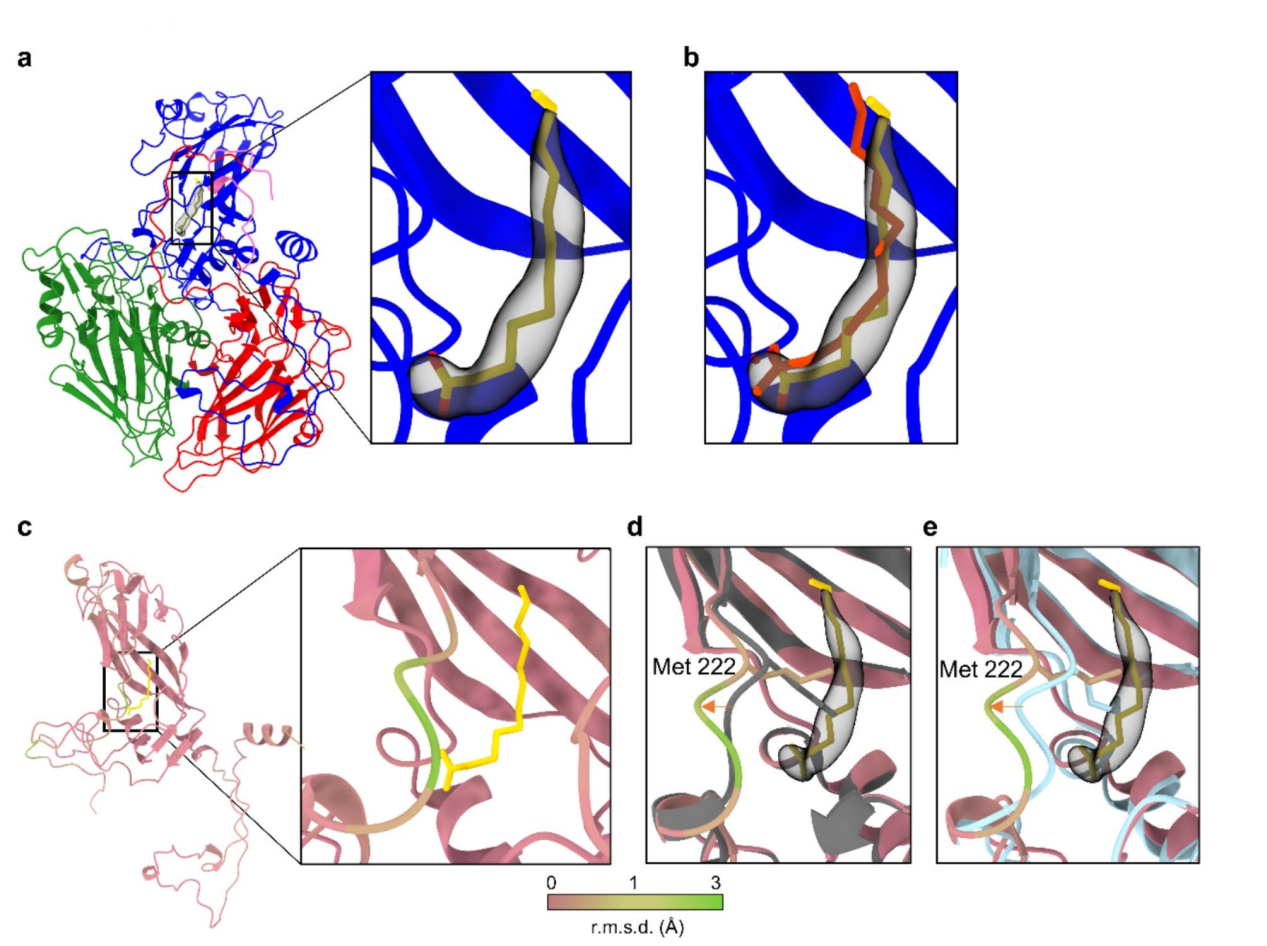
In the absence of DMSO myristate occupies the VP1 binding pocket. (**a**) Atomic model of one RV-A89 protomer with cryo-EM density (shown in grey) inside of the VP1 binding pocket identified as myristate (shown in yellow). (**b**) Comparison of the position of the myristate in the VP1 binding pocket of RV-A89 capsid (shown in yellow) with myristate (shown in red) in the hydrophobic pocket of RV-A16 (PDB 1AYM). Full protomers were superimposed using ChimeraX software matchmaking functionality. (**c**) Atomic model of full RV-A89 VP1 protein colored by alpha-carbons r.m.s.d. R.m.s.d was calculated from our atomic model and the previously published model derived from the cryo-EM structure of RV-A89 imaged in the presence of 10% DMSO (PDB: 6SK7) using ChimeraX (same r.m.s.d. coloring used in (**d**,**e**)). (**d**,**e**) Atomic model comparison of VP1 binding pockets derived from 3D cryo-EM reconstruction in absence (colored by r.m.s.d values) and (**d**) presence (colored in grey) of DMSO or (**e**) the empty particle (colored in light blue). Orange arrow indicates the movement of the GH loop away from the binding pocket in the presence of myristate (shown in yellow).

Using the remaining 80% of the particles we solved the structure of the native RV-A89 to a near atomic resolution of 1.76 Å, locally ranging between 1.55 Å and 1.85 Å. Overall, the high quality of the map allowed us to build an atomic model including 189 structured water molecules (Fig. 1d, e; Sup. Figure 1a–f). Thorough analysis of the hydrophobic pocket revealed an isolated density with elongated shape, which cannot be assigned to a protein chain; it is at the assumed position of a myristate and has its typical shape. Although the full density of the fatty acid appears better visible in a low pass filtered (2.5 Å) map, the density is sharp enough to determine the correct position and orientation of the molecule (Fig. 2a). The fact that the myristate could not be clearly seen at higher resolution suggests either partial occupancy across the icosahedral virion (i.e. less than the 60 positions would be occupied) and/or loose binding to the hydrophobic pocket. The latter would explain why DMSO readily elutes myristate from its binding site, or why antiviral drug candidates can easily displace the myristate as shown previously for many rhinovirus types and antivirals^24^. Finally, to confirm its correct position as well as its orientation we compared our atomic model containing myristate with those of other RVs obtained earlier by x-ray crystallography or cryo-EM (Fig. 2b)^9^.

To understand the implication of the missing myristate on the viral conformation, we analyzed the differences of the two atomic models, derived from cryo-EM maps reconstructed from data acquired in the presence or absence of DMSO. First, we determined the average root mean squared deviation (r.m.s.d.Ø) of the alpha-carbons between each pair of VP 1–3 chains. This analysis revealed a low average r.m.s.d. (r.m.s.d.Ø 0.653 Å, 0.355 Å, 0.410 Å and 0.486 respectively) for each VP chain (Supp. Figure 1g); this shows that the overall structure of individual VP chains remained mainly identical. However, focused r.m.s.d. analysis of residues forming the hydrophobic pocket revealed a significant conformational change of a five amino acid stretch at the end of the G-H loop corresponding to VP1 residues 218–223 with local average r.m.s.d.Ø of 3.692 Å (Fig. 2c). By superimposing both atomic models we observed that in the DMSO-containing buffer the pocket was empty and collapsed; the side chain of VP1-Met222 had a different conformation resulting in it partially filling the void, whereas in the native structure the 6 amino acid stretch is significantly re-arranged and moved away with respect to the hydrophobic pocket providing space for the myristate (Fig. 2d, Supp. Figure 1h). For completeness, we also analyzed the structure of the reconstructed empty capsid (the B particle); it also exhibits an empty and collapsed hydrophobic pocket missing the myristate; similarly to the native empty structure, residue VP1-Met222 reaches into the pocket (Fig. 2e). This agrees with previously published structural data of uncoated particles^20^. Finally, based upon our structural data we conclude that incubation of RV-A89 with DMSO elutes the myristate from the hydrophobic pocket and renders the conformation of the pocket more like the pocket of the uncoated virion. This agrees with the idea of a three-step process of uncoating: (1) exit of the pocket factor, (2) exit of VP4, and (3) exit of the RNA.

### The cryo-EM map of RV-A89 in plain buffer is highly symmetric, including the interfaces between individual protomers and the RNA

Previously, using the RV-A89 structure obtained in the presence of DMSO by using 3D-classification of computationally extracted single protomers, different conformations of the amino acid residues in contact with the asymmetric RNA were found at the interfaces of the sixty individual, symmetrically related RV-A89 subunits^11^. The author speculated that this might reflect the different folds of the asymmetric RNA molecule leading to distinct interactions of identical amino acid side chains with the different nucleotide sequences and folds of the RNA genome^11^. Whereas the icosahedral viral protein shell is highly symmetric, the RNA genome is not. This necessarily requires the RNA’s different sequences and their 3D-conformations to interact with identical amino acid side chains of each of the sixty protomers of the protein capsid.

**Fig. 3:**
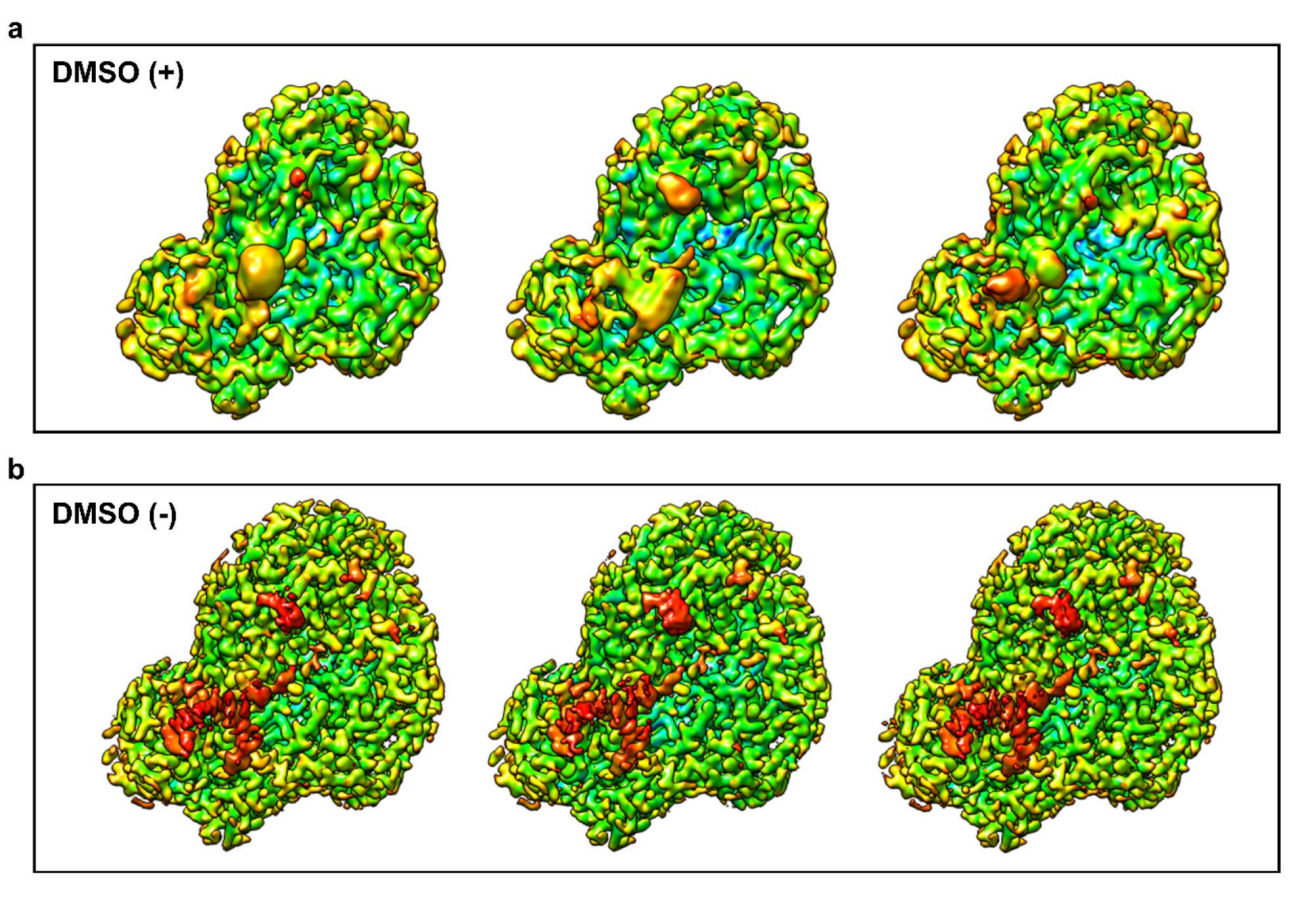
DMSO and/or the lack of the pocket factor accentuate asymmetry in the RNA interaction sites on the inner surface of the RV-A89 capsid. (**a**) 3D classification into three classes of single protomers computationally extracted from icosahedrally expanded datasets of RV-A89 in 10% DMSO (image taken from ref^11^, modified) and (**b**) of RV-A89 in plain buffer. The procedure used for the “plain buffer” data set was the same as specified in ref^11^. Colored from green to red according to Chimera’s volume data gradient norm. View from inside the capsid towards the outside. Note the striking differences in the density at the interface to the RNA (red) between the three classes in the sample containing DMSO and the virtual identical conformation found within the classes lacking DMSO. Also note the difference in resolution of the viral maps used (2.9 Å for the DMSO + data set vs. 2.1 Å for the DMSO- dataset).

Aiming at benefiting from the higher resolution of the map obtained from the new data set, we repeated the above 3D-classification by the same procedure as used previously. Surprisingly, these analyses, exemplified for three 3D-classes (Fig. 3a), showed no appreciable difference between the classes, as becomes obvious from a comparison with previous data (Fig. 3b; i.e. Figure 3 from ref. (11) modified). We tentatively explain this finding by assuming that the stabilization of the virion by the pocket factor^25–29^ counteracts movements necessary for adaptation of the amino acid side chain conformations to the different RNA 3D-structures.

In summary, we have demonstrated that use of DMSO as a solvent for hydrophobic compounds might elute low affinity ligands from their hydrophobic binding sites and impact RNA protein interactions. These significant effects should be kept in mind when carrying out interaction and stability assays in the presence of considerably high concentrations of this solvent.

## Discussion

One of the major immunodominant sites on the surface of *Enteroviruses* is located on the G-H loop of VP1. The end of the G-H loop has been identified as a conformationally highly dynamic area, forming part of a hydrophobic pocket created under main contribution of the VP1 protein. In native virions, this hydrophobic pocket is filled with naturally occurring ‘pocket factors’. These hydrophobic compounds are believed to enhance the stability of the viral capsid. The 3D structures of some RVs, e.g. of RV-B14, were found to lack this pocket factor, suggesting that it was lost during sample preparation. This loss might be reversible, as suggested by our coincidental observation that addition of myristate to purified virions can increase their stability (data not shown). This is in line with results on related *Enteroviruses*; which show that incubation with fatty acid-free albumin (and endosomal ions), presumably leading to extraction of the pocket factors, resulted in destabilization and addition of fatty acids led to stabilization^30,31^. The pocket factors have been implicated in the regulation of viral entry, uncoating, and assembly^9^. Small organic molecules target this specific area by substituting the natural pocket factors and significantly increase stability of the GH loop and, thereby, of the entire capsid^14^. Similarly, our observations further support the hypothesis that the reversible interaction of these naturally occurring factors with the VP1 pocket may serve as a subtle regulatory mechanism for the conformational dynamics of the GH loop, directly impacting the processes of viral entry and uncoating.

In this study, the structure of RV-A89 studied in plain buffer contains myristate in its hydrophobic pocket. The fact that in the presence of OBR-5-340 and DMSO the ligand is absent the binding pocket collapses and an increased structural flexibility at the protein-RNA interface is observed, suggests that virus stiffening is a consequence of filling the hydrophobic pocket with either natural ligand or with antivirals^32–36^. Furthermore, DMSO, even at low concentrations, can modify RNA-ligand interactions and affinity^12^. Thus, the above-described structural changes in RV-A89 further confirm the potential of DMSO to induce changes in protein and viral capsid structures, specifically through regions with hydrophobic binding sites occupied by natural factors. These may alter the conformation and stability of the proteins and interfere with functional dynamics and could result in incorrect assumptions about the shape, flexibility, or accessibility of potential drug-binding sites, thus impacting subsequent drug design. In drug screening, such distortions could mask true binding interactions, like water molecules, or imply drug affinities that do not reflect the natural state. In conclusion, our findings add further evidence that DMSO might affect the structural integrity of proteins and, more broadly, viral capsids^37–40^.

## Materials and methods

The RV-A89 sample was prepared as described previously^14^. Four microliters of purified RV-A89 at 5 mg/ml in 50 mM MgCl_2_, 50 mM Tris-HCl (pH 7.4) was applied onto glow discharged (30 s, 25 mA) gold Quantifoil grids (2/2 300 mesh), coated with a thin layer (1 nm) of amorphous carbon (made in-house). After application, followed by incubation for 45 s, the sample was plunge-frozen in a propane: ethane (63:37) mixture at liquid nitrogen temperature using a Vitrobot Mark V (ThermoFisher Scientific) set to 100% humidity and 4 °C. Vitrified samples were imaged on a Titan Krios TEM (ThermoFisher Scientific) operating at 300 kV, equipped with a field emission gun (XFEG) and a Gatan Bioquantum energy filter with a slit of 10 eV and a Gatan K3 electron detector. In total 4,859 movies were recorded in electron-counting super-resolution mode at 105,000x nominal magnification (physical pixel size 0.826 Å, super-resolution pixel size 0.413 Å at the specimen level) consisting of 40 frames over 2 s (total electron exposure of 44 e^−^ Å^−2^, corresponding to 1.1 e^−^ Å^−2^ per frame) using ThermoFisher Scientific EPU data collection software using a defocus range between − 0.2 and − 1 μm.

### Cryo-EM image processing and atomic model building

Single-particle analyses were performed using Relion^41^ (v4.0-beta2 and v5) and cryoSPARC^42^ (4.4.1). Movies were motion-corrected using MotionCor2^43^ (implemented in Relion), dose-weighted (using 1.1 e^−^ Å^−2^ per frame) and the contrast transfer function (CTF) parameters were estimated with CTFFIND4^44^ (v4.1.14). Particles were automatically picked from the motion-corrected micrographs by using crYOLO^45^ (v1.4) trained with a subset of manually picked particles. Viral particles were extracted at 1000 × 1000 px and subjected to several rounds of 2D classification to separate full and empty viral capsids and re-extracted. Using the previously determined RV-A89 volume as initial model, several rounds of 3D-refinement, per-particle CTF and Bayesian polishing were performed in Relion. Finally, particles were imported in cryoSPARC and subjected to Homo-refinement followed by Local-refinement. Local resolution estimates, gold-standard resolution (Fourier shell correlation = 0.143) and sharpened maps were calculated by using cryoSPARC (Table 1).

**Table 1:**
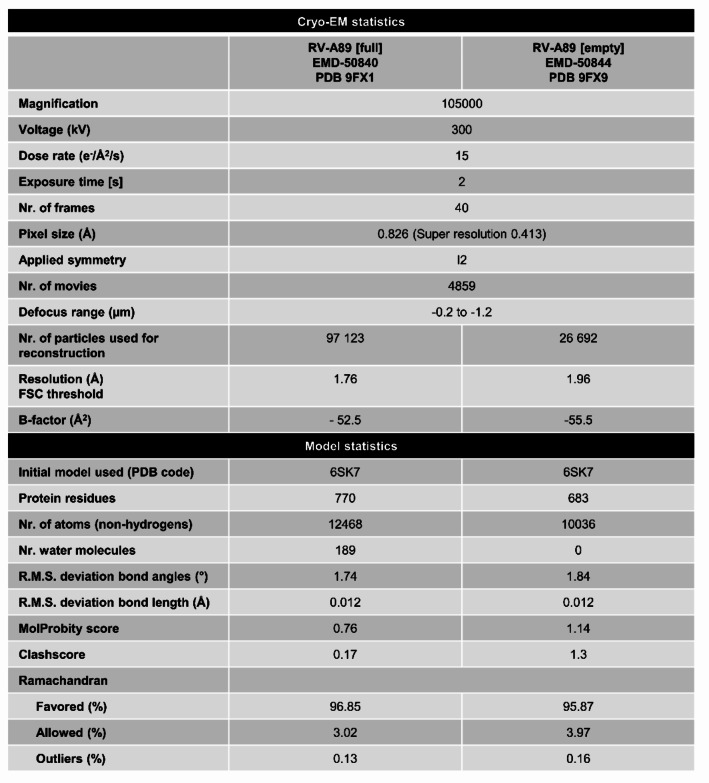
Cryo-EM data collection, model refinement, and validation statistics.

Model building was started by fitting the PDB 6SK7 model into electron microscopy maps using the fit-in-map tool in UCSF Chimera^46^ (v1.17). Initial model refinements were done using Rosetta^47^ (v3.13) controlled via StarMap^48^ (v1.2.15). Further interactive refinement was performed with ISOLDE^49^ within UCSF ChimeraX^50^ (v1.6.1). Water molecules were automatically added using Phenix.douse and coordinate files were finally refined with Phenix.real_space_refine^51^ (v1.20.1–4487) using reference model restraints, strict rotamer matching and disabled grid search settings. The MolProbity^52^ server was used to validate model geometries. UCSF Chimera (1.13 and 1.17) and ChimeraX^50^ (v1.6.1) were used for visualizations and analysis. Using ViperDB^53^ tools dimensions and net surface charges for “full” and “empty” virions were determined.

## Electronic supplementary material

Below is the link to the electronic supplementary material.

Supplementary Material 1

## Data Availability

Cryo-EM density maps resolved in this study have been deposited in the Electron Microscopy Data Bank (EMDB) (www.emdataresource.org) under accession codes: EMD-50840 and EMD-50844. The corresponding coordinates have been deposited in the Protein Data Bank (PDB) (https://www.pdb.org) under accession codes: 9FX1, 9FX9.
