# Supplementary material for: DMSO might impact ligand binding, capsid stability, and RNA interaction in viral preparations
Source: Sci Rep. 2024 Dec 6;14:30408. doi: 10.1038/s41598-024-81789-x (PMC11621809; doi:10.1038/s41598-024-81789-x)
Supplement: Supplementary file 1 — Supplementary Material 1 [file 41598_2024_81789_MOESM1_ESM.docx]

**
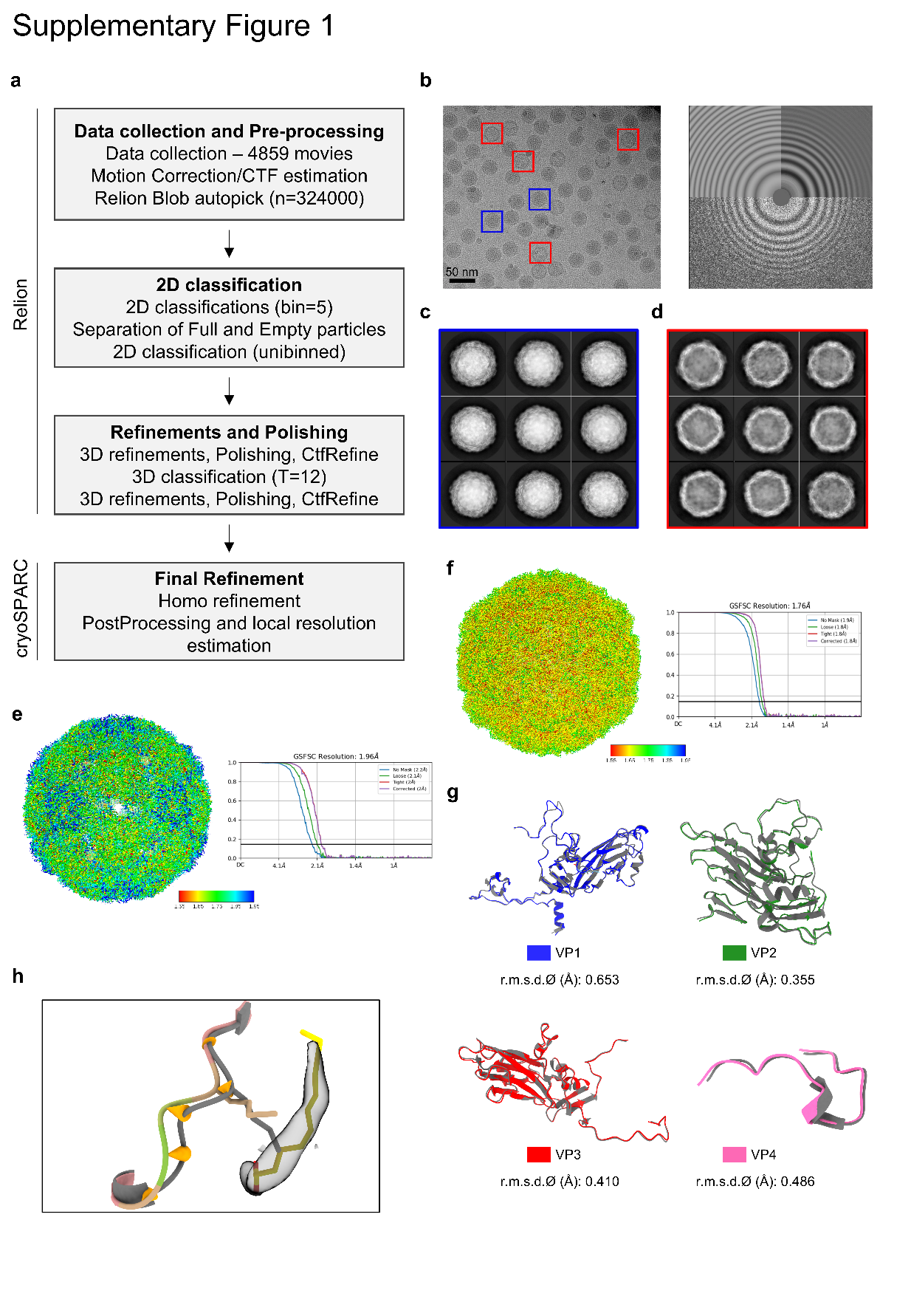
**

**Supplementary Figure 1.** (a) Cryo-EM single particle data processing workflow of HRV-A89. (b) Representative cryo-EM micrograph and (c-d) selected 2D classes indicating full (blue) and empty (red) RV-A89 particles. (e) Local resolution estimations and Fourier Shell Correlation (FSC) plots of the I2 of the RV-A89 empty and (f) full 3D reconstructions. (g) Superposition of VP1-4 proteins (shown in ribbon) originating from DMSO+ (in grey) and DMSO- (colored) with corresponding averaged r.m.s.d values (in Å). (h) Atomic model comparison of VP1 residues 218 - 223 in binding pockets derived from 3D cryo-EM reconstruction in absence (colored by r.m.s.d values) and presence (colored in grey) of DMSO. Orange arrows indicate the magnitude and directionality of the GH loop movement between matching Cα-atom pairs away from the binding pocket in the presence of myristate (shown in yellow).
